# Evaluation of industrial *Saccharomyces cerevisiae* strains as the chassis cell for second-generation bioethanol production

**DOI:** 10.1111/1751-7915.12245

**Published:** 2015-01-23

**Authors:** Hongxing Li, Meiling Wu, Lili Xu, Jin Hou, Ting Guo, Xiaoming Bao, Yu Shen

**Affiliations:** 1 State Key Laboratory of Microbial Technology, Shandong UniversityJinan, 250100, China; 2 Guangzhou Sugarcane Industry Research InstituteGuangzhou, 510316, China

## Abstract

To develop a suitable *Saccharomyces cerevisiae* industrial strain as a chassis cell for ethanol production using lignocellulosic materials, 32 wild-type strains were evaluated for their glucose fermenting ability, their tolerance to the stresses they might encounter in lignocellulosic hydrolysate fermentation and their genetic background for pentose metabolism. The strain BSIF, isolated from tropical fruit in Thailand, was selected out of the distinctly different strains studied for its promising characteristics. The maximal specific growth rate of BSIF was as high as 0.65 h^−1^ in yeast extract peptone dextrose medium, and the ethanol yield was 0.45 g g^−1^ consumed glucose. Furthermore, compared with other strains, this strain exhibited superior tolerance to high temperature, hyperosmotic stress and oxidative stress; better growth performance in lignocellulosic hydrolysate; and better xylose utilization capacity when an initial xylose metabolic pathway was introduced. All of these results indicate that this strain is an excellent chassis strain for lignocellulosic ethanol production.

## Introduction

Biofuels (including ethanol, butanol and biodiesel), chemicals and other commodities produced from renewable and abundant lignocellulosic feedstocks have become increasingly important because of the depletion of fossil fuel energy sources and growing public concerns about the environment and food security (Zhou *et al*., 2012; Jönsson *et al*., 2013). One of the most practical solutions is to produce bioethanol from lignocellulosic feedstocks with *Saccharomyces cerevisiae* (Palmqvist and Hahn-Hägerdal, 2000a). However, this natural ethanol producer faces several new challenges when the substrate is lignocellulose instead of starch. Not only a high glucose metabolism capacity and ethanol yield, but also the capacity to tackle the challenges associated with lignocellulose fermentation are necessary properties for a lignocellulosic ethanol-producing strain.

The lignocellulosic ethanol conversion process generally includes raw material pretreatment, cellulose hydrolysis, sugar fermentation by microorganisms and distillation. Most of the hemicellulose fraction is hydrolysed to monosaccharides in the pretreatment step, and the xylose obtained during this process is the second most abundant sugar in lignocellulosic materials (Kim *et al*., 2013). Efficient use of xylose would greatly increase the economic benefits of lignocellulosic bioethanol production (Sun and Cheng, 2002; Hahn-Hägerdal *et al*., 2007). However, xylose is not naturally fermented by *S. cerevisiae*. The inability of *S. cerevisiae* to use xylose is not only due to its lack of relevant enzymes, but also related to the low efficiency of other necessary metabolic pathways, such as the pentose phosphate pathway. In addition, inhibitors that are formed during the pretreatment and hydrolysis processes with the release of sugars are toxic to microorganisms. Therefore, the yeast strain used in lignocellulosic bioethanol production requires not only high ethanol yields from both glucose and xylose, but also robustness in its harsh working environment.

The inhibitors to ethanol production are usually divided into three major groups: weak acids, furan derivatives and phenolic compounds (Palmqvist and Hahn-Hägerdal, 2000b; Almeida *et al*., 2007; Alriksson *et al*., 2010). The inhibitory effects of these compounds on *S. cerevisiae* strains are quite different. Undissociated weak acids are liposoluble and can diffuse across the plasma membrane, causing intracellular anion accumulation and inhibiting cell growth. Furan derivatives, such as furfural and HMF (5-hydroxymethylfurfural), have been shown to reduce the specific growth rate, the cell mass yield, and the volumetric and specific ethanol productivities. Phenolic compounds destroy the cell membrane integrity, thereby impeding the membrane's function (Palmqvist and Hahn-Hägerdal, 2000b; Almeida *et al*., 2007). The interaction effects of these inhibitor compounds often lead to more serious inhibition (Palmqvist *et al*., 1999). In addition, some hydrolysate components that have not been extensively analysed also cause undetermined stresses on the cells (Palmqvist and Hahn-Hägerdal, 2000b). In addition to the osmotic stress caused by the ions and sugars in the hydrolysate, the ethanol product also has negative effects on yeast (Sonderegger *et al*., 2004; Jönsson *et al*., 2013).

Metabolic and evolutionary engineering strategies have been extensively performed in constructing and enhancing the xylose fermentation capacity of both laboratory and industrial *S. cerevisiae* strains (Eliasson *et al*., 2000; Kuyper *et al*., 2003; Peng *et al*., 2012; Zhou *et al*., 2012). The fermentation properties and inhibitor resistance depend on the strains' individual genetic backgrounds. The diploid or polyploid industrial *S. cerevisiae* strains are usually more robust than the haploid strains, and the whole-genome duplication in yeast was proposed to lead to an efficient fermentation system (Piškur *et al*., 2006). The choice of the chassis strain has a significant effect on the performance of lignocellulosic hydrolysate-based ethanol production (Brandberg *et al*., 2004), and it is important to possess a self-owned, suitable chassis strain because of intellectual property concerns.

Therefore, in this paper, 32 wild-type *S. cerevisiae* strains were evaluated. Among them, candidate strains with an ethanol yield of more than 0.41 g g^−1^ consumed glucose were selected in the preliminary screening. Then, the tolerance to inhibitors, the growth performance in hydrolysate and the background xylose utilization capacity of the candidate strains were further compared. In addition, the ploidy of the strains was determined. Based on these evaluations, an *S. cerevisiae* strain was chosen as the chassis cell for constructing a lignocellulosic ethanol-producing strain.

## Results and discussion

### Collection of *S. cerevisiae* strains

In this work, 32 wild-type *S. cerevisiae* strains were collected or isolated from several strain preservation institutions, ethanol production companies, commercial active dry yeasts and different habitats. The detailed strain sources and numbers are listed in Table 1. The strains isolated from specific environments were identified by sequence analysis of the 26S rDNA D1/D2 domain.

**Table 1:** *Saccharomyces cerevisiae* strain sources and numbers used in this work

Strain sources	Strain number	Strains evaluated^a^
Obtained from ATCC, CICC, CGMCC	6	CICC31034
Used in starch-based ethanol production	6	NAN-27, 6508
Isolated from commercially available active dry yeast (China, Sweden, America, France, Japan)	5	
Isolated from grape or wine production regions	4	RC212
Isolated from liquor production regions	3	
Isolated from tropical fruit in Thailand	3	BSIF
Isolated from waste materials in sugar cane industry	5	

a The five strains selected for all evaluations and their corresponding sources.

ATCC, American Type Culture Collection; CGMCC, China General Microbiological Culture Collection Center; CICC, China Center of Industrial Culture Collection.

### Fermentation performance of *S. cerevisiae* strains on glucose

**Figure 1:**
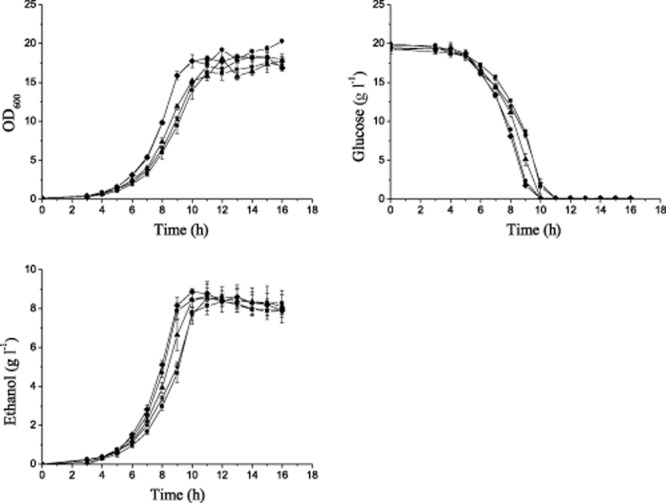
Oxygen-limited fermentation on glucose in shake flasks. The cells were cultured at 30°C in shake flasks with initial OD_600_ of 0.1 (∼ 0.02 g l^−1^ dry cell biomass). Oxygen-limited conditions were obtained with a rubber stopper and a syringe needle, and the cells were agitated at 200 r.p.m. The experiments were performed in triplicate. Symbol: *S. cerevisiae* strain NAN-27, ▪; BSIF, ♦; RC212, ▴; CICC31034, •; 6508, ⋆.

Efficient ethanol production from glucose, a main component in lignocellulosic hydrolysate, is the most important characteristic for the candidate strains for bioethanol production. Therefore, the fermentation performances of the *S. cerevisiae* strains were first evaluated with glucose as the sole carbon source. The fermentation was performed in shake flasks with an initial cell density of OD_600_ 0.1 (∼ 0.02 g l^−1^ dry cell biomass). Rubber stoppers with syringe needles were used for oxygen-limited cultivations. As expected, these strains exhibited significant differences in the ethanol yield, ranging from 0.33 to 0.43 g g^−1^ consumed glucose after 10 h. Five strains, NAN-27, BSIF, RC212, CICC31034 and 6508 (Table 1), with ethanol yields of more than 0.41 g g^−1^ consumed glucose, were selected and evaluated in further detail. The results in Fig. 1 and Table 2 show that strains BSIF and RC212 had the best performance in terms of the maximum specific growth rate (μ_max_) and corresponding glucose volumetric consumption rate and that the ethanol yield of both strains was higher than 0.44 g g^−1^ consumed glucose.

**Table 2:** Metabolic characteristics of *S. cerevisiae* strains on glucose in shake flasks

Strains	μ_max_ (h^−1^)	Ethanol yield (g g^−1^ consumed glucose)^a^	Glucose volume consumption rate (g l^−1^ h^−1^)^b^
NAN27	0.590 ± 0.005	0.421 ± 0.003	1.183 ± 0.106
BSIF	0.652 ± 0.004	0.451 ± 0.016	1.966 ± 0.055
RC212	0.632 ± 0.005	0.441 ± 0.001	1.939 ± 0.021
CICC31034	0.609 ± 0.006	0.423 ± 0.007	1.653 ± 0.071
6508	0.556 ± 0.022	0.425 ± 0.001	1.238 ± 0.048

a Ethanol yield was calculated at the time of maximal ethanol concentration determined by high-performance liquid chromatography (HPLC).

b Glucose volume consumption rate was calculated at the fermentation time of 9 h.

### Evaluation of tolerance of *S. cerevisiae* strains to individual stress factors

**Figure 2:**
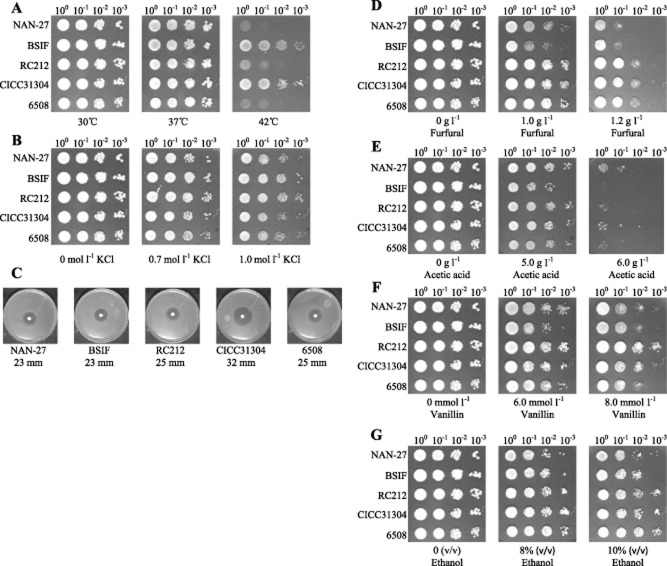
Growth of *S. cerevisiae* strains on the plate at stress conditions. (A) Temperatures; (B) osmotic stress with high concentration of KCl; (C) oxidative stress with H_2_O_2_; (D) furfural; (E) acetic acid; (F) vanillin; (G) ethanol. Except for measuring the oxidative stress, 4 μl of each suspension from 10-fold serial dilution with initial OD_600_ of 1.0 was spotted onto the plates. For oxidative stress (C), approximately 100 μl OD_600_ 2.0 cells were mixed with 20 ml agar, and 6 μl 30% H_2_O_2_ was dropped in the centre of the 0.5 mm diameter sterile filter paper. All of the cell populations were incubated at 30°C for 2 days, but two more temperatures, 37°C and 42°C, were considered for thermotolerance evaluation.

Considering the stress conditions that the fermentation strain may encounter in the second-generation fuel ethanol production process, the growth performances of the five selected strains were tested under aerobic conditions with several stress factors (Fig. 2). The performances of the five *S. cerevisiae* strains were greatly different. Strains BSIF and CICC31034 clearly grew better at a higher temperature, 42°C (Fig. 2A). The hypertonic stress tolerance of BSIF and RC212 was slightly higher than the tolerance of other strains on plates with 1.0 mol^−1^ KCl (Fig. 2B). Strain CICC31034 was more sensitive to oxidative stress by 30% H_2_O_2_ than the other four strains (Fig. 2C). The tested strains showed distinct tolerance to the inhibitors in the hydrolysate, but no single strain showed the highest tolerance to all of the different inhibitors. Strain NAN-27 showed more tolerance to acetic acid (Fig. 2E) but less to furfural (Fig. 2D) and vanillin (Fig. 2F), strains RC212 and CICC31034 grew better on vanillin plates (Fig. 2F). All of the tested strains exhibited strong ethanol tolerance, all growing well in 10% ethanol (Fig. 2G). The results suggested the relative robustness of strains; however, under anaerobic fermentation conditions, the specific tolerance of strains to stresses might be lower because of a lower energy supply.

### Evaluation of tolerance of *S. cerevisiae* strains to corn stover hydrolysate

**Figure 3:**
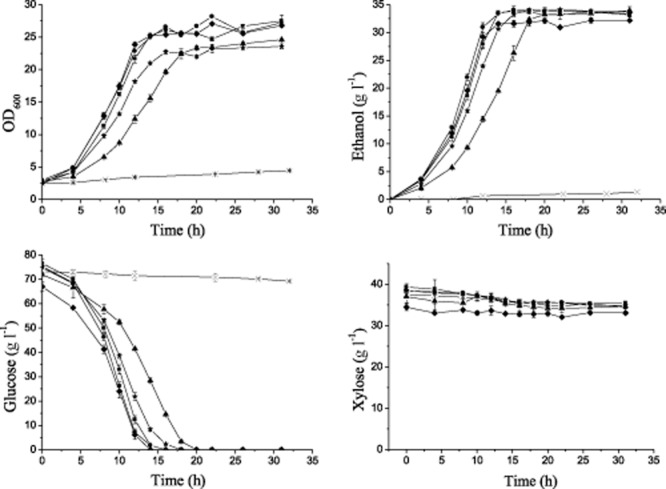
Oxygen-limited fermentation in corn stover hydrolysate. The cells were cultured at 30°C in shake flasks with initial OD_600_ of 2.5 (∼ 0.5 g l^−1^ dry cell biomass), and the pH was adjusted to 6.0 with 5 mol l^−1^ NaOH. Oxygen-limited conditions were obtained with a rubber stopper and a syringe needle, and the cells were agitated at 200 r.p.m. The experiments were performed in triplicate. Symbol: *S. cerevisiae* strain NAN-27, ▪; BSIF, ♦; RC212, ▴; CICC31034, •; 6508, ⋆; CEN.PK 113-5D, ×.

Lignocellulosic hydrolysate is the strains' working medium for second-generation bioethanol production. In this hydrolysate, weak acids, furan derivatives and phenolic compounds are generally recognized as major toxicants, and their mixture usually increases their inhibitory impact (Almeida *et al*., 2007). Furthermore, there are other toxic substances not readily identified in hydrolysate (Palmqvist and Hahn-Hägerdal, 2000b). The fermentation characteristics of the five industrial strains and the laboratory strain CEN.PK 113-5D were therefore tested in hydrolysed corn stover. The hydrolysate contained 3.7 g l^−1^ acetic acid, 0.39 g l^−1^ furfural and 0.29 g l^−1^ HMF in addition to sugars. The total phenolics and solubilized lignin in the hydrolysate were 15.5 mmol l^−1^ and 4 g l^−1^ respectively (Table 3). The *S. cerevisiae* strains were inoculated into this hydrolysate (the pH was adjusted to 6.0 with 5 mol l^−1^ NaOH) with an initial cell density of OD_600_ 2.5 (∼ 0.5 g l^−1^ dry cell biomass). Shake flasks with rubber stoppers, and syringe needles were used for oxygen-limited cultivations. As expected, the growth and fermentation of the auxotrophic haploid laboratory strain CEN.PK 113-5D was strongly inhibited. All five of the wild-type strains exhibited good fermentation characteristics in the hydrolysate, with strains BSIF and NAN-27 showing the best characteristics (Fig. 3). These strains consumed almost all of the glucose but no xylose because of the absence of enzymes converting the xylose to xylulose (Peng *et al*., 2012).

**Table 3:** Main components in corn stover hydrolysate

Components		Concentration
Monosaccharides (g l^−1^)	Glucose	77.06 ± 0.63
	Xylose	29.32 ± 0.24
	Galactose	3.34 ± 0.05
	Arabinose	5.29 ± 0.02
	Mannose	0.79 ± 0.04
Weak acids (g l^−1^)	Formic acid	ND
	Acetic acid	3.72 ± 0.02
	Levulinic acid	ND
Furan aldehydes (g l^−1^)	Furfural	0.39 ± 0.01
	5-HMF	0.29 ± 0.01
Total phenolics (mmol l^−1^)		15.51 ± 0.86
Solubilized lignin (g l^−1^)		3.99 ± 0.07

ND, not detected.

### Evaluation of inherent capacity for xylose utilization

**Figure 4:**
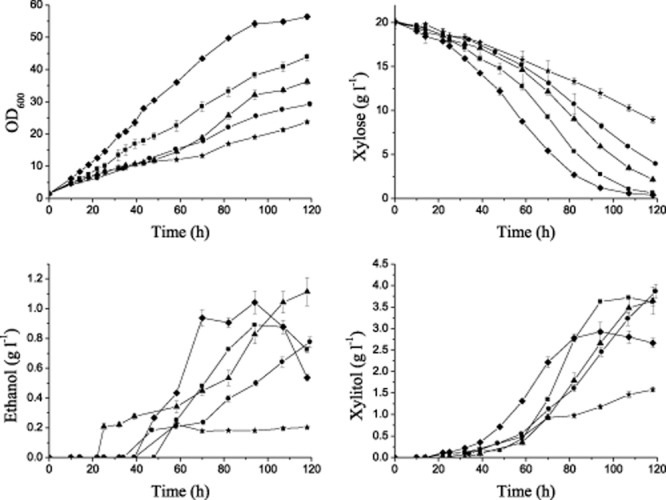
Oxygen-limited fermentation on xylose in shake flasks. The cells were cultured at 30°C in shake flasks with initial OD_600_ of 1.5(∼ 0.3 g l^−1^ dry cell biomass). The oxygen-limited conditions were obtained with a rubber stopper and a syringe needle, and the cells were agitated at 200 r.p.m. The experiments were performed in triplicate. The plasmid pYMIK-xy127, containing genes of reductase, xylitol dehydrogenase and xylulokinase (Wang *et al*., 2004), was integrated into the chromosome. Symbol: *S. cerevisiae* strain NAN-127, ▪; BSIF-127, ♦; RC212-127, ▴; CICC31034-127, •; 6508-127, ⋆.

It is generally considered that *S. cerevisiae* cannot metabolize xylose. However, some strains have weak background xylose utilization (Toivari *et al*., 2004) or show different xylulose fermentation characteristics (Matsushika *et al*., 2009). A stronger xylose or xylulose metabolic background is an advantage to constructing a glucose and xylose co-utilizing strain. Our strains could not utilize xylose, as mentioned above, and xylulose is expensive and difficult to prepare. Therefore, for selecting a strain with a better capacity for using xylose, a basic initial xylose metabolic pathway was introduced into the chromosomes of the candidate strains using the plasmid pYMIK-xy127 (Wang *et al*., 2004), which contains the *Scheffersomyces (Pichia) stipitis* genes *XYL1* (encoding xylose reductase), *XYL2* (encoding xylitol dehydrogenase) and the *S. cerevisiae* gene *XKS1* (encoding xylulokinase). Then, their inherent capacity for xylulose utilization was evaluated using YEPX (yeast extract-peptone xylose) medium (10 g l^−1^ yeast extract, 20 g l^−1^ peptone and 20 g l^−1^ xylose). Among the recombinant strains, strain BSIF with the addition of the *XYL1-XYL2-XKS1* showed the highest maximum specific growth rate (μ_max_) (0.060 h^−1^) on xylose and highest xylose volumetric consumption rate (0.201 g l^−1^ h^−1^) (Fig. 4 and Table 4), suggesting a better inherent capacity for xylose utilization. Because no more modifications had been made on the recombinant strains, low ethanol yields were obtained because of the accumulation of xylitol. However, less xylitol was accumulated in BSIF-127 than in the other strains except 6508-127, which used only a small quantity of xylose. This result suggested that the BSIF strain has a more flexible cofactor pool than other strains.

**Table 4:** Metabolic characteristics of recombinant *S. cerevisiae* strains on xylose in shake flasks

Strains	μ_max_ (h^−1^)	Ethanol yield (g g^−1^ consumed xylose)^a^	Xylose volume consumption rate (g l^−1^ h^−1^)^b^
NAN-127	0.055 ± 0.001	0.052 ± 0.004	0.183 ± 0.023
BSIF-127	0.060 ± 0.003	0.064 ± 0.009	0.201 ± 0.031
RC212-127	0.037 ± 0.002	0.063 ± 0.003	0.151 ± 0.018
CICC-127	0.030 ± 0.000	0.048 ± 0.008	0.124 ± 0.026
6508-127	0.033 ± 0.002	0.051 ± 0.010	0.087 ± 0.038

a Ethanol yield was calculated at the time of maximal ethanol concentration determined by high-performance liquid chromatography (HPLC).

b Xylose volume consumption rate was calculated at the fermentation time of 94 h.

### Ploidy confirmation of *S. cerevisiae* strains

**Figure 5:**
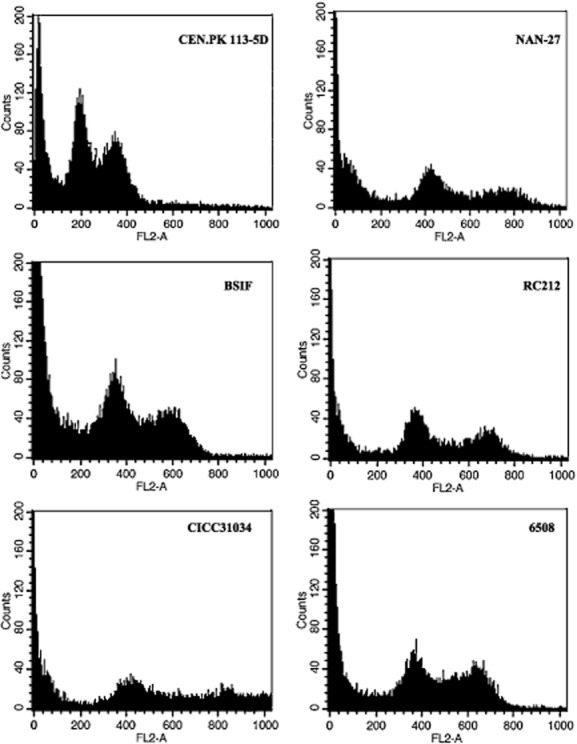
Determination of DNA content by flow cytometry of *S. cerevisiae* strains. Strains were grown to exponential phase then fixed with ethanol, and the DNA was stained with propidium iodide. The x-coordinate FL2-A represents total cell fluorescence, and it is proportional to DNA content. Two Gaussian curves in density plot represent the G0/G1 and G2/M phase of cells respectively. The strain ploidy was then determined by the G0/G1 peak value on FL2-A versus that of the haploid strain, which was set as 200.

Keeping the characteristics obtained by molecular modification stable is one of the most important issues for industrial use. Moreover, the molecular modifications of wild-type industrial strains are generally performed on their chromosomal DNA. Therefore, it is necessary to determine the ploidy of the industrial strain. The strain ploidy was determined by the G0/G1 peak value on FL2-A versus that of the haploid strain CEN.PK 113-5D, which was set as 200. The results showed that all of the strains we evaluated were diploid or triploid. The five *S. cerevisiae* strains we selected were all diploid as shown by their G0/G1 peak values on FL2-A of nearly 400, which was about twice that of the control haploid strain (Fig. 5).

The performances of the five strains are summarized in Table 5. Strain BSIF was isolated from tropical fruit in Thailand, RC 212 was selected from a wine production region, and NAN-27 (Wang *et al*., 2004; Zhang *et al*., 2010) and 6508 are currently being used in first-generation bioethanol production in China. Among the five strains, BSIF showed superior properties for most of the evaluated characteristics and was better than the first-generation bioethanol strains NAN-27 and 6508.

**Table 5:** The summary of growth or fermentation performances and ploidy of the *S. cerevisiae* strains.^a^

Line		NAN-27	BSIF	RC212	CICC31304	6508
1	Oxygen limited fermentation on glucose	+	+++	+++	++	+
2	High temperature	+	+++	+	+++	+
3	Hyper-osmotic stress	+	+++	+++	++	++
4	Oxidative stress	+++	+++	++	+	++
5	High concentration of ethanol	++	++	+++	+++	+++
6	Furfural stress	+	+	++	++	++
7	Acetic acid stress	++	+	++	++	++
8	Vanillin stress	++	++	+++	++	+
9	Growth performance in hydrolysate	+++	+++	+	+++	++
10	Potential xylose transport and downstream metabolism capacity	++	+++	+	+	+
11	Ploidy	Diploid	Diploid	Diploid	Diploid	Diploid

a The greater the number of ‘+’ symbols, the better the growth or fermentation performance.

Second-generation bioethanol production faces more complex challenges than producing ethanol from starch. To produce lignocellulose bioethanol in a cost-effective manner, the *S. cerevisiae* strain needs to utilize multiple types of monosaccharides, especially glucose and xylose, under various stresses arising from the inhibitors in the lignocellulosic substrate. However, the regulation mechanism of stress tolerance is very complex and not very well understood. Choosing a strain with naturally suitable properties as a chassis is more convenient than conferring these properties on it by engineering. In the present study, we give an example of a systematic evaluation to select a suitable chassis strain for lignocellulosic ethanol production. Because of its high ethanol yield from sugar, good growth capacity in hydrolysate, better potential xylose utilization capacity and superior tolerance to most of the stress factors, strain BSIF was selected, and more genetic modifications and evolutionary work will be implemented on it to produce cost-effective lignocellulosic bioethanol.

## Experimental procedures

### The preculture of *S. cerevisiae* strains

Thirty-two wild-type *S. cerevisiae* strains from different sources were used in the present study (Table 1). A single colony from an agar plate was cultured in YEPD medium (10 g l^−1^ yeast extract, 20 g l^−1^ peptone and 20 g l^−1^ glucose) overnight at 30°C. The culture was then transferred into 250 ml shake flasks containing 100 ml YEPD medium at an initial OD_600_ of 0.2 and incubated for 12 h at 30°C. Then, the precultured cells were harvested and used as inoculants.

### Identification of yeast strains

The genomic DNA of the yeast strains was extracted as described previously (Hong *et al*., 2007a). The sequences of the rDNA D1/D2 domain were amplified and sequenced (Hu *et al*., 2012). The wild-type yeast strains were identified using the sequence analysis of the 26S rDNA D1/D2 domain.

### Stress tolerance assays

The growth performance of selected *S. cerevisiae* strains under the typical stresses of furfural, acetic acid, vanillin and ethanol, as well as hyper-osmotic stress (Chen, 1989; Liu *et al*., 2006), oxidative stress (Stephen *et al*., 1995; Liu *et al*., 2006) and high temperature (Hong *et al*., 2007b; Abdel-Banat *et al*., 2010) were characterized through a spot dilution growth assay. Cells were harvested from an overnight culture in YEPD liquid medium and washed twice with sterile water. The density of re-suspended cells was normalized to an OD_600_ of 1.0. A 10-fold serial dilution of this suspension (10^0^, 10^−1^, 10^−2^ and 10^−3^) was prepared, and 4 μl of each dilute suspension was spotted onto the appropriate solid medium (Liu *et al*., 2009).

To determine the oxidative stress resistance of each strain, *S. cerevisiae* cells were washed with 100 μl sterile water and re-suspended. The density was adjusted to OD_600_ 2.0, and the cells were mixed with 20 ml YEPD agar (cooled to approximately 50°C) and immediately poured into a plate. Then, a sterile filter paper (0.5 mm diameter) with 6 μl 30% H_2_O_2_ was placed in the centre of each plate. The oxidative stress resistance of each strain was demonstrated by the diameter of the zone of growth inhibition (mm) after cultivation for 2 days at 30°C. A smaller inhibition zone was interpreted as a higher resistance to oxidative stress (Liu *et al*., 2006).

### The construction of xylose-fermenting recombinant strains

An integrated plasmid, pYMIK-xy127 (Wang *et al*., 2004), containing the *Scheffersomyces (Pichia) stipitis* genes *XYL1* (encoding xylose reductase) and *XYL2* (encoding xylitol dehydrogenase) and the *S. cerevisiae* gene *XKS1* (encoding xylulokinase) as described previously was linearized with *Hpa*I and then transformed into the *S. cerevisiae* strains using the lithium acetate method. Colonies of recombinant cells were selected on YEPD plates containing 0.4 g l^−1^ of the antibiotic G418 (Wang *et al*., 2004).

### Batch fermentation

All of the batch fermentations were performed in 50 ml of medium in 100 ml flasks with rubber stoppers (with a syringe needle to release CO_2_ during fermentation) to maintain the oxygen-limited conditions, and the flasks were shaken at 200 r.p.m. To determine the fermentation performance using glucose, the cells were cultured in YEPD medium with an initial cell density of OD_600_ 0.1 (∼ 0.02 g l^−1^ dry cell biomass). To evaluate tolerance to hydrolysate, the cells were cultured in hydrolysate with 5 g l^−1^ ammonium sulfate added, and the initial cell density was adjusted to OD_600_ 2.5 (∼ 0.5 g l^−1^ dry cell biomass). To evaluate the potential xylose utilization capacity, the cells were cultured in YEPX medium (10 g l^−1^ yeast extract, 20 g l^−1^ peptone and 20 g l^−1^ xylose) with an initial OD_600_ 1.5 (∼ 0.3 g l^−1^ dry cell biomass).

### Analytical methods

Biomass was measured by a turbidity determination at 600 nm. For the analysis of metabolites, culture samples taken at different time points were first centrifuged at 13 000 r.p.m. for 15 min, and then the supernatants were filtered through a 0.45 μm membrane. The concentrations of glucose, xylose, xylitol, glycerol, acetate and ethanol were determined using HPLC (Shimadzu, Japan) with a BIO-RAD Aminex HPX-87H ion exclusion column (300 × 7.8 mm) and a refractive index detector (RID-10A). The mobile phase was 5 mmol l^−1^ H_2_SO_4_ with a flow rate of 0.6 ml min^−1^. The temperature of the column oven was 45°C (Zhang *et al*., 2010). To analyse the monosaccharides in the hydrolysate, a BIO-RAD Aminex HPX-87P ion exclusion column (300 × 7.8 mm) was used at 80°C with a mobile phase of water at a flow rate of 0.6 ml min^−1^ (Wang *et al*., 2013). The weak acids, furfural and HMF in the hydrolysate were measured using the HPX-87H column as mentioned above. Total phenolics were determined using the Folin phenol method, and vanillin was used to prepare the standard curve (Singleton *et al*., 1999). Solubilized lignin was determined by measuring the absorbance at 320 nm (Tan *et al*., 2013).

### Ploidy determination of *S. cerevisiae* strains

The ploidy of the *S. cerevisiae* strains was determined by fluorescently labelling the cell nuclei and then analysing the fluorescence of each cell in the population by flow cytometry. The haploid strain CEN.PK 113-5D was used as a reference strain. Samples of *S. cerevisiae* cells (2 × 10^6^ cells ml^−1^) were fixed in 70% (v/v) ethanol (–20°C) and kept at –20°C for 2 h. Then, the cells were washed twice with PBS (137 mmol l^−1^ NaCl, 2.7 mmol l^−1^ KCl, 10 mmol l^−1^ phosphate buffer, pH = 7.4) and re-suspended in 1 ml PBS containing 1 mg ml^−1^ RNAase A (Sigma). After 2 h of incubation at 37°C, cells were washed with PBS and re-suspended in 200 μl of PBS containing 50 μg ml^−1^ propidium iodide. Data were collected on a linear scale. Under these conditions, the fluorescence is proportional to the DNA content (Santos *et al*., 2013).

## Conflict of interest

None declared.
